# Supplementary figures and images for: CellProfiler 4: improvements in speed, utility and usability
Source: BMC Bioinformatics. 2021 Sep 10;22:433. doi: 10.1186/s12859-021-04344-9 (PMC8431850; doi:10.1186/s12859-021-04344-9)

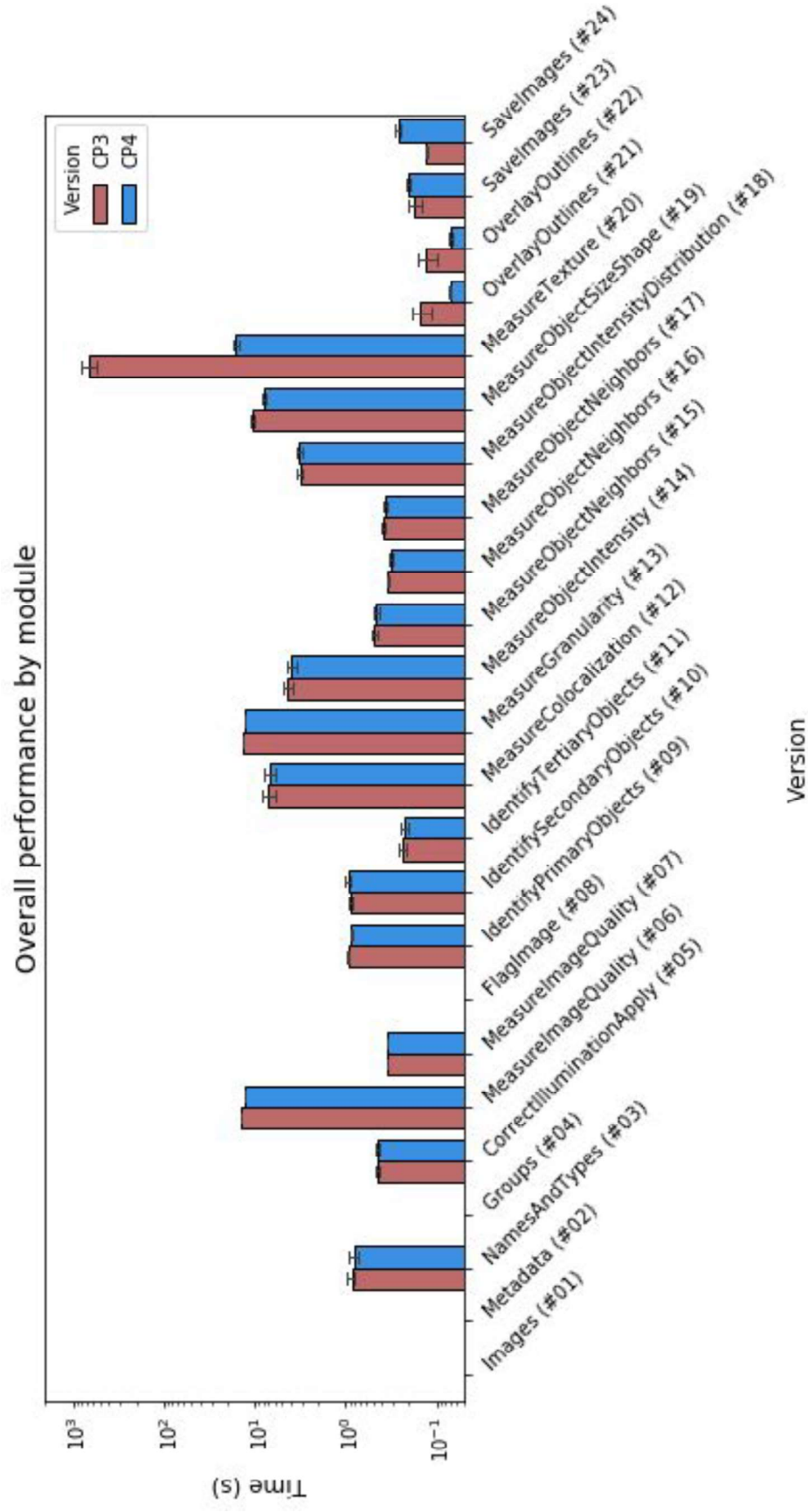

Figure S1

Supplement: Supplementary file 1 — Additional file 1: Figure S1. Execution times of all modules within the Cell Painting example pipeline. Measured as per-image CPU time taken for each module in the Cell Painting assay protocol (n = 48). I/O loading operations in the Images module are not recorded by these measurements. [file 12859_2021_4344_MOESM1_ESM.pdf]
